# Single-Incision Multiport/Single Port Laparoscopic Abdominal Surgery (SILAP): A Prospective Multicenter Observational Quality Study

**DOI:** 10.2196/resprot.5557

**Published:** 2016-09-07

**Authors:** Rene Mantke, Markus Diener, Siegfried Kropf, Ronny Otto, Thomas Manger, Boris Vestweber, Lutz Mirow, Günther Winde, Hans Lippert

**Affiliations:** ^1^Brandenburg Medical SchoolDepartment of SurgeryUniversity Hospital Brandenburg / HavelBrandenburgGermany; ^2^2Study Centre of the German Surgical Society (SDGC)Department of General, Visceral and Transplantation SurgeryUniversity of Heidelberg, GermanyHeidelbergGermany; ^3^University Hospital of MagdeburgInstitute for Biometry and Medical InformaticsOtto von Guericke UniversityMagdeburgGermany; ^4^Institute for Quality Assurance in Surgical CareInstituteOtto von Guericke UniversityMagdeburgGermany; ^5^SRH HospitalDepartment of General, Visceral and Pediatric SurgerySRH Wald HospitalGeraGermany; ^6^City HospitalDepartment of SurgeryHospital LeverkusenLeverkusenGermany; ^7^HospitalDepartment of SurgeryHeinrich Braun HospitalKirchbergGermany; ^8^HospitalDepartment of SurgeryHospital HerfordHerfordGermany

**Keywords:** single-incision, quality study, minimally invasive

## Abstract

**Background:**

Increasing experience with minimally invasive surgery and the development of new instruments has resulted in a tendency toward reducing the number of abdominal skin incisions. Retrospective and randomized prospective studies could show the feasibility of single-incision surgery without any increased risk to the patient. However, large prospective multicenter observational datasets do not currently exist.

**Objective:**

This prospective multicenter observational quality study will provide a relevant dataset reflecting the feasibility and safety of single-incision surgery. This study focuses on external validity, clinical relevance, and the patients’ perspective. Accordingly, the single-incision multiport/single port laparoscopic abdominal surgery (SILAP) study will supplement the existing evidence, which does not currently allow evidence-based surgical decision making.

**Methods:**

The SILAP study is an international prospective multicenter observational quality study. Mortality, morbidity, complications during surgery, complications postoperatively, patient characteristics, and technical aspects will be monitored. We expect more than 100 surgical centers to participate with 5000 patients with abdominal single-incision surgery during the study period.

**Results:**

Funding was obtained in 2012. Enrollment began on January 01, 2013, and will be completed on December 31, 2018. As of January 2016, 2119 patients have been included, 106 German centers are registered, and 27 centers are very active (>5 patients per year).

**Conclusions:**

This prospective multicenter observational quality study will provide a relevant dataset reflecting the feasibility and safety of single-incision surgery. An international enlargement and recruitment of centers outside of Germany is meaningful.

**Trial Registration:**

German Clinical Trials Register: DRKS00004594; https://drks-neu.uniklinik-freiburg.de/drks_web/navigate.do?navigationId=trial.HTML&TRIAL_ID=DRKS00004594 (Archived by WebCite at http://www.webcitation.org/6jK6ZVyUs)

## Introduction

The surgical standard for many abdominal diseases is changing. Traditionally, operative treatments meant open resection with laparotomy. However, over the last two decades, laparoscopic surgery has become a valuable alternative to many procedures, for example, cholecystectomy, appendectomy, or colon resection [1-6].

Conventional laparoscopic surgery requires a number of ports and a specimen extraction incision. Increasing experience with minimally invasive surgery and the development of new instruments has resulted in a tendency toward reducing the number of skin incisions. Natural orifice transluminal endoscopic surgery (NOTES) is the closest we have come to scar-free surgery because it does not leave any visible scars on the surface of the body. But NOTES is still an experimental approach [7]. For that reason, single-incision laparoscopic surgery could represent an attractive approach to minimally invasive abdominal surgery [8]. Several recently published retrospective and randomized prospective studies could demonstrate that the single-incision single port/multiport technique is not associated with a higher rate of morbidity or mortality compared with conventional laparoscopic surgery [9-21]. The majority of the relevant literature documents single-incision cholecystectomy. Single-incision appendectomy, liver surgery, colon surgery, and gastric surgery have also been described [22-28].

The determination of differences in the safety of classic multitrocar techniques compared to the single-incision techniques in prospective randomized studies is still difficult because of the rarity of some relevant complications. For example, injury to the major bile duct during surgery, with a frequency of about 0.5%, is a rare but severe complication. Large prospective randomized studies with many thousands of subjects are necessary to answer some of these clinically relevant questions; however, such large prospective randomized studies are not necessarily feasible. Prospective multicenter observational quality studies should support the data of published prospective randomized studies on this subject.

While surgeons develop new techniques for entering the abdominal cavity, patient safety should remain important. This prospective multicenter observational quality study will provide a relevant dataset reflecting the feasibility and safety of single-incision surgery. This study focuses on external validity, clinical relevance, and the patient perspective. Accordingly, the single-incision multiport/single port laparoscopic abdominal surgery (SILAP) study will supplement the existing evidence, which is currently too sparse to allow evidence-based surgical decision making.

## Methods

### Trial Objectives

The aim of the trial is to collect data about indications, technical aspects, mortality, and morbidity of single-incision multiport or single port abdominal surgery in daily use. All types of single-incision multiport or single port abdominal surgery will be included in the study. Every participating center will receive a quality report every year (online in real time for the individual center and in a print version once a year for the complete study). The analysis time period for the annual quality report is one calendar year. This quality report will be available for each center by May of the ongoing year and will include data from the previous year.

### Trial Design

SILAP is a prospective nonrandomized multicenter observational quality study with no intervention.

### Trial Duration and Schedule

**Figure 1:**
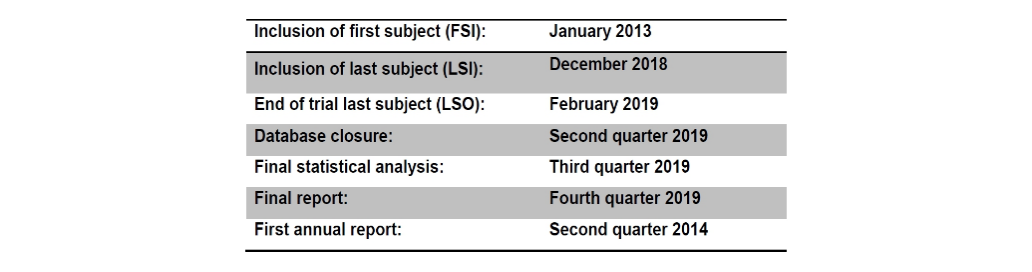
Trial duration.

The duration of the trial for each patient is limited to the hospital stay. The trial duration itself is from January 2013 to December 2018 (see Figure 1). The actual overall duration or recruitment may vary.

### Study Population

**Figure 2:**
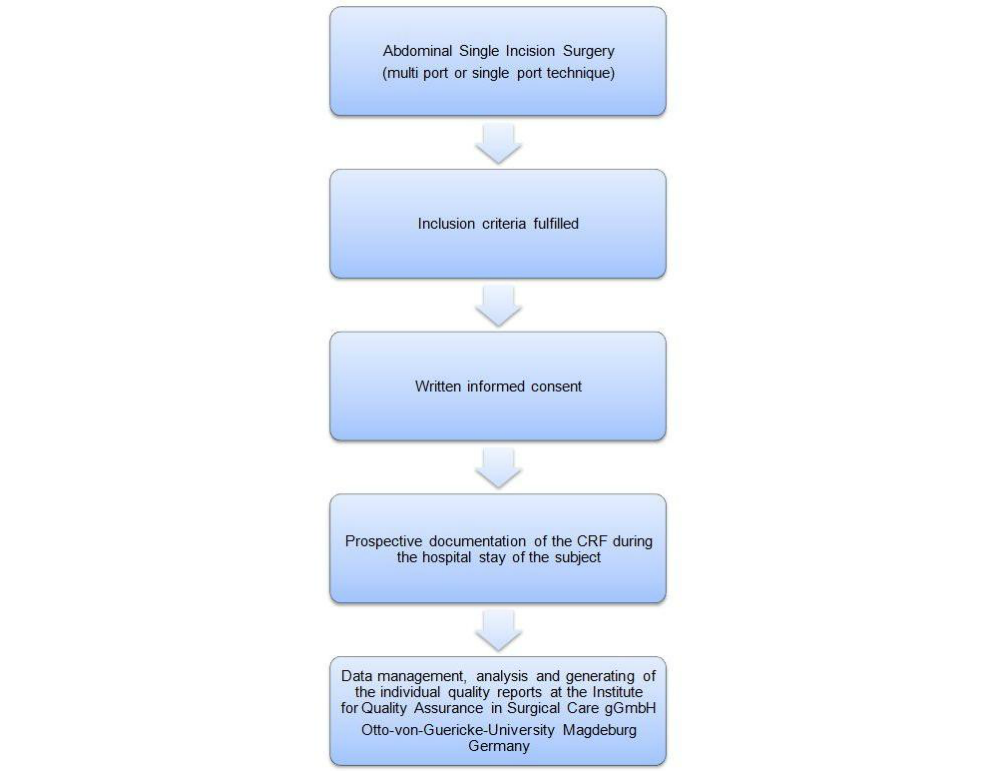
Trial flow chart.

All patients at the participating centers who are scheduled for single-incision multiport or single port abdominal surgery will be informed about the purpose of the observational trial. All types of abdominal surgery can be included. After being screened for the inclusion and exclusion criteria, eligible patients will be included in the trial (informed consent necessary) (see Figure 2). Surgery, examinations, and measures are not influenced by this observational trial.

### Number of Subjects and Trial Centers

A sample size calculation is not relevant for this observational quality study. A minimum of 100 surgical centers is expected. The participating centers will include a maximum of their patients fulfilling the inclusion criteria and not meeting an exclusion criterion. The data are documented using an Internet database including a self-plausibility control function.

### Inclusion Criteria

Subjects meeting all of the following criteria will be considered for admission to the trial: (1) patients (≥18 years) scheduled for elective or emergency single-incision multiport or single port abdominal surgery (all types of abdominal surgery), (2) ability of subject to understand character of the clinical trial, and (3) able and willing to provide written informed consent.

### Exclusion Criteria

Subjects presenting with the following criteria will not be included in the trial: participation in another intervention trial that interferes with the intervention and outcome of this study.

### Outcome Variables

The following endpoints will be used to answer the trial goals: (1) Mortality (hospital mortality), (2) Morbidity, which includes complications during surgery (eg, bleeding during surgery or injury of small or large bowel, common bile duct, urine bladder, ureter, solid organs, or other intraabdominal structures) and post-operative complications (eg, wound infection according to Centers for Disease Control and Prevention [CDC] criteria, intraabdominal infections, urinary tract infection, pneumonia, cardiac complications, pulmonary complications, intraabdominal bleeding after surgery, ileus, insufficiency of an anastomosis, reoperation, burst abdomen, or other complications), (3) Patient characteristics (sex, age, height in cm, weight in kg, duration of hospital stay, duration of hospital stay after surgery, routine or emergency surgery, date of surgery, American Society of Anesthesiologists classification [29], indication only for cholecystectomy), and (4) Technical aspects, such as type of surgery (ie, appendectomy or cholecystectomy), type of single-incision procedure (multiport or single port), location of the single incision (umbilical or other position), used port device or size and number of the trocars for single-incision surgery, success rate of the single-incision procedure (procedure finished in single-incision technique), incision to closure time (operation time), conversion rate, device for closing the cystic duct, use of holding sutures or other holding devices, and type of sutures for closing the fascia incision.

**Figure 3:**
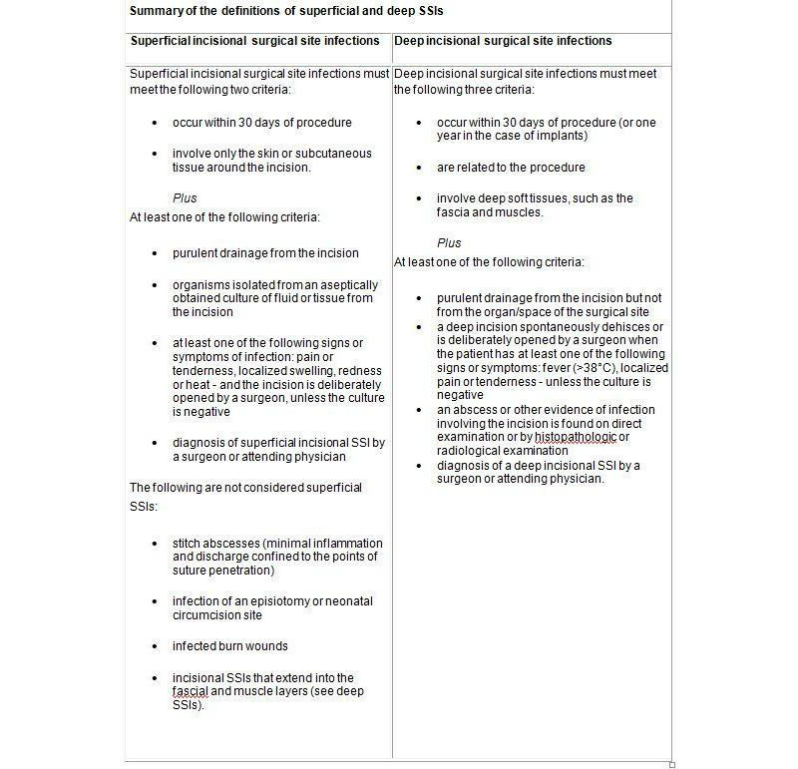
Definitions of superficial and deep SSIS (adapted from Horan TC et al).

The assessment of outcome variables comprises (1) mortality, that is, death due to any cause at any time during the hospital stay, and (2) morbidity (during the hospital stay), including:

- surgical site infections (SSIs) will be assessed at discharge and divided into superficial and deep incisional SSIs according to the CDC definition [30] (see Figure 3)
- post-op pulmonary infection (pneumonia) will be assessed at discharge and is defined as infection of the lung with either evidence of increased infection parameters (C-reactive protein/CRP >2mg/dl and/or leukocytes >10 0000/ml), which are not caused by a different pathologic process, or evidence of pulmonary infiltration in the chest x-ray, requiring antibiotic therapy
- lesion of small or large bowel (any unplanned complete lesion of bowel)
- bleeding during surgery (any bleeding during surgery that required conversion to an open approach or to an multiple trocar approach or required transfusion)
- intraabdominal bleeding after surgery (requiring transfusion or surgery)
- intraabdominal infections (any kind of peritonitis, intraperitoneal abscess, infected bilioma requiring medication, drainage, or surgery)
- ileus (any kind of postoperative intestine passage disturbance requiring medication or surgery)
- insufficiency of an anastomosis (every anastomotic leak clinical or radiographic proven)
- burst abdomen (postoperative dehiscence of the fascia/peritoneum with or without dehiscence of the skin)

Other aspects for assessment are operation time (from skin incision to closure of wound), conversion rate (conversion from single incision to an open approach), and need for additional trocars.

### Statistical Procedures

Every participating center will get an annual report detailing their cases. The documented case report form data will be described in relationship to the study population. Continuous variables will be represented with the usual international metrics: mean, standard aberration, minimum, lower quartile, mean, median, higher quartile, and maximum. Categorical variables will be represented with absolute and relative frequencies.

The overall description of the whole study population will be completed with subgroup analyses according to important patient characteristics (eg, gender and age) or technical aspects (eg, type of surgery). The influence of potential prognostic factors on the outcome variables will be studied in logistic regression analyses at an exploratory level.

### Ethics

The procedures set out in a trial protocol pertaining to the conduct, evaluation, and documentation of this trial are designed to ensure that all persons involved in the trial abide by good clinical practice and the ethical principles described in the current revision of the Declaration of Helsinki. The trial will be carried out in accordance with local legal and regulatory requirements. The trial protocol, the informed consent document, and any other appropriate documents were accepted by the Ethics Committee of the Otto von Guericke University of Magdeburg (#148/12). Since all surgical procedures examined in this trial are well established and in current daily use, no increased medical risks are expected for the participating patients. This study is registered with the German Clinical Trials Register (DRKS00004594).

## Results

Funding was obtained in 2012; enrollment began on January 01, 2013 and will be completed on December 31, 2018. As of January 2016, 2119 patients have been included, 106 German centers are registered, and 27 centers are very active (>5 patients per year).

## Discussion

### Principle Considerations

Extensive literature exists on laparoscopic abdominal surgery. Conventional laparoscopic surgery requires a number of ports and a specimen extraction incision. Surgeons all over the world try to minimize abdominal wall trauma by using single-incision surgery. Single-incision surgery can use a single port system or a single-incision multiport approach [31-34]. Retrospective and randomized prospective studies could show the feasibility of this type of surgery without any increased risk to the patient. Of course, patients are usually selected and surgeons are very experienced. This limits the power of these studies. Some studies show advantages for single-incision surgery in postoperative pain, cosmetics, and patient satisfaction [35-39]. However, studies focused on pain were not blinded, which could lead to a relevant bias. Operative time was usually longer for the single-incision single port/multiport techniques [40]. Advantages of single-incision laparoscopic surgery, for example, reduced risk of hemorrhage or hernia, are intuitive at this time. The previous randomized prospective studies have too few patients to answer important questions in this area because of the low frequency of some relevant complications. For instance, it is still unclear if single-incision surgery leads to an increase in common bile duct injuries. To answer this question in a prospective randomized study is not easy or feasible, because there would be a need for thousands of patients to be included.

### Conclusion

The endpoints of this study will allow a comprehensive evaluation of the surgical technique of single-incision abdominal laparoscopic surgery and its results. The results of this prospective trial will be compared with available evidence (ie, data from prospective randomized studies or reviews of single-incision surgery and classic multiple trocar techniques) in order to substantiate the current knowledge in this area.

## Abbreviations

CDC: Centers for Disease Control and Prevention
NOTES: natural orifice transluminal endoscopic surgery
SILAP: single-incision multiport/single port laparoscopic abdominal surgery study
SSI: surgical site infection
